# Is Institutional Delivery Protective Against Neonatal Mortality Among Poor or Tribal Women? A Cohort Study From Gujarat, India

**DOI:** 10.1007/s10995-016-2202-y

**Published:** 2016-12-30

**Authors:** Rebecca Altman, Kristi Sidney, Ayesha De Costa, Kranti Vora, Mariano Salazar

**Affiliations:** 1 Department of Public Health Sciences, Karolinska Institutet, Stockholm, Sweden; 2 Department of Reproductive and Child Health, Indian Institute of Public Health, Gandhinagar, Ahmedabad, Gujarat India

**Keywords:** Maternal complications, Childbirth, Neonatal mortality, Institutional delivery, India

## Abstract

*Objectives* In low-income settings, neonatal mortality rates (NMR) are higher among socioeconomically disadvantaged groups. Institutional deliveries have been shown to be protective against neonatal mortality. In Gujarat, India, the access of disadvantaged women to institutional deliveries has increased. However, the impact of increased institutional delivery on NMR has not been studied here. This paper examined if institutional childbirth is associated with lower NMR among disadvantaged women in Gujarat, India. *Methods* A community-based prospective cohort of pregnant women was followed in three districts in Gujarat, India (July 2013–November 2014). Two thousand nine hundred and nineteen live births to disadvantaged women (tribal or below poverty line) were included in the study. Data was analyzed using multivariable logistic regression. *Results* The overall NMR was 25 deaths per 1000 live births. Multivariable analysis showed that institutional childbirth was protective against neonatal mortality only among disadvantaged women with obstetric complications during delivery. Among mothers with obstetric complications during delivery, those who gave birth in a private or public facility had significantly lower odds of having a neonatal death than women delivering at home (AOR 0.07 95% CI 0.01–0.45 and AOR 0.03, 95% CI 0.00–0.33 respectively). *Conclusions for Practice* Our findings highlight the crucial role of institutional delivery to prevent neonatal deaths among those born to disadvantaged women with complications during delivery in this setting. Efforts to improve disadvantaged women’s access to good quality obstetric care must continue in order to further reduce the NMR in Gujarat, India.

## Significance

*What is already known on this subject?* The protective effect that institutional childbirth has on neonatal mortality varies between countries and depends on the type of health facility (public or private) accessed. In India, it is not clear whether institutional childbirth is protective against neonatal mortality among children born to vulnerable tribal or poor women who carry the burden of neonatal mortality. *What this study adds?* Private (AOR 0.07, 95% CI 0.01–0.45) or public (AOR 0.03, 95% CI 0.00–0.33) institutional childbirth was protective against neonatal mortality only among poor/tribal women with obstetric complications during childbirth.

## Introduction

Despite significant declines in under-five mortality globally, progress has been inadequate towards achieving Millennium Development Goal (MDG) 4–a two-thirds reduction in under-5 mortality by 2015 (You et al. 2014). While mortality across all age cohorts under-five has declined, headway has been slowest in reduction global neonatal mortality rates (NMR) (Lawn et al. 2010). The proportion of neonatal mortality as a share of global child under-five mortality has increased from 37 to 44% between 1990 and 2013 (You et al. 2014).

Even though India has experienced a 43% decline in neonatal mortality between 1990 and 2013, the country still accounts for more than a quarter of global neonatal deaths (28%) with a NMR of 29 per 1000 live births in 2013 (Wardlaw et al. 2014). In 2013, India recorded the highest absolute number of neonatal deaths of any country, (748,000) (Wardlaw et al. 2014).

As in many low and middle-income settings, the burden of neonatal mortality in India is concentrated among socioeconomically disadvantaged groups (Dogra et al. 2015; Lawn et al. 2005, 2010; Paul et al. 2011; Ram et al. 2013). For example, a population-based study in three Indian states found that 64% of neonatal deaths occurred among below poverty line (BPL) mothers (Dogra et al. 2015).

### Preventing Neonatal Mortality

Up to two-thirds of global neonatal deaths are preventable through simple interventions and health systems strengthening (Lawn et al. 2005). Community and facility-based interventions have been shown to be effective to reduce neonatal mortality (Darmstadt et al. 2005). For example, the World Health Organization’s Integrated Management of Childhood Illness is an approach that combines home-based detection and treatment of illness with improved in-facility care (World Health Organization 2003). Furthermore, it has been argued that the risk of neonatal mortality can be reduced by 29% in low- and middle-income countries if birth occurs in a health facility, because of quality and safety of care assumed possible during in-facility deliveries in low-resource settings (Tura et al. 2013). Additional characteristics of institutional childbirth that contribute to the quality and safety of deliveries compared to home births include access to skilled birth attendants, medical equipment and referral structures (Tura et al. 2013). Evidence from a population-based study in China showed that, compared to babies born at home, those born in hospitals had a 48–70% lower risk of neonatal death (Feng et al. 2011). However, this protective effect varies significantly between countries, and depends on the type of facility accessed (Fink et al. 2015). An analysis of demographic surveys in 67 low- and middle-income countries found that only delivery in a private facility was protective against early neonatal mortality compared to delivering at home (Fink et al. 2015).

### Institutional Delivery in Asia

Demographic surveys from five countries in Asia reported an increase of 10–20 percentage points in the proportions of institutional deliveries during the period of 1995–2005 (Limwattananon et al. 2011). Institutional childbirth in India has also steadily increased from 38% in 2005 to 74% in 2013 (De Costa et al. 2014). Similar increases have occurred in the Indian state of Gujarat (De Costa et al. 2014).

Epidemiological studies mapping the magnitude (Ram et al. 2013), causes (The Million Death Study 2006) and determinants of neonatal mortality (Kumar et al. 2014; Rammohan et al. 2013) have been conducted in India. However, these studies have not explored if institutional childbirth is protective against neonatal mortality among vulnerable women (defined here as being BPL or tribal) who carry the burden of neonatal mortality in India. Thus, this population-based cohort study aims to assess whether institutional delivery protects against neonatal mortality among vulnerable populations (tribal or BPL) in Gujarat state, India.

## Methods

### Study Setting

The state of Gujarat on India’s western flank has a population of 60.4 million (Bharadwaj 2011). It is divided into 26 administrative units called districts, each with a population of 1–1.5 million people (Bharadwaj 2011). Despite its rapid economic growth, 57.4% of the population lives in rural settings, 17% is BPL (Bharadwaj 2011) and 14% is tribal (Scheduled Tribe-ST) (Ministry of Social Justice and Empowerment 2005). BPL status is assigned by the Indian government to families with earning below a certain threshold. It indicates economic disadvantage and allows access to government aid. Scheduled Tribes (ST) are historically disadvantaged indigenous social groups who are recipients of positive affirmative action after India’s independence in 1947 and listed under Article 366 (25) of the Indian constitution (Government of Gujarat 2009). As of 2012, the NMR in Gujarat was 28/1000, similar to the Indian national average of 29/1000 (Zodpey and Paul 2014).

The provision of obstetric services (intrapartum care) in Gujarat lies both in the public and private sector, but is dominated by the private sector (De Costa et al. 2014). Over the last decade, there have been a number of state led programs to increase the proportion of institutional delivery among poor/tribal women. Institutional delivery in the state rose from 40% in 2001 to 89% of all births in 2010, approximately 60% of which occur in the private sector (De Costa et al. 2014).

### Study Design and Sampling Frame

This community-based prospective cohort study is nested within the MATIND project (Sidney et al. 2012) a comprehensive study of programs designed to reduce financial access barriers that preclude women from obtaining emergency obstetric care. This project was implemented in three districts (Dahod, Sabarkantha, and Surendranagar) in Gujarat, which were purposively selected to cover diverse geographic and socioeconomic areas of the state (Sidney et al. 2012). The characteristics of the district are described in Table 1.

**Table 1:** District characteristics

District	Total population	Rural (%)	Average literacy (%)
Dahod	2,127,086	91	58.8
Sabarkantha	2,428,589	85	75.8
Surendranagar	1,756,268	72	72.1

Source: Districts of Gujarat 2011

http://www.census2011.co.in/census/state/districtlist/gujarat.html

Participating mothers and their newborns were identified through two-stage cluster sampling. First, villages with a population of between 1000–2500 (of which at least 40% were BPL according to 2001 government census) were identified. There were 142 such villages in the three study districts.

**Fig. 1:**
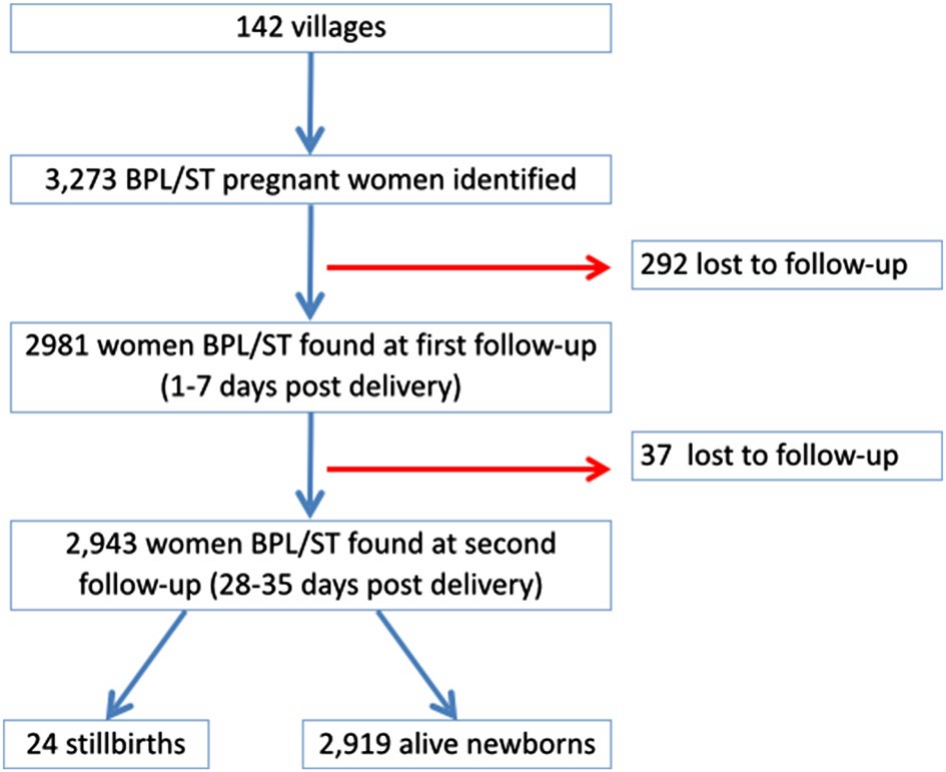
Sample flow chart

Within a selected village, a survey of all households (facilitated by local village health workers) was conducted to identify pregnant women in the 3rd trimester. These women were contacted and an initial interview was done by trained researchers to elicit information on their socio-demographic, antenatal care and expected delivery dates. Subsequently, two home follow-ups with the mothers were conducted. The first follow-up occurred 1–7 days post-delivery and the second at 28–35 days post-delivery. The purpose of the first follow-up was to gather birth data, the occurrence on any intra partum complications, early neonatal morbidity and mortality. This allowed us to reduce recall bias. The second follow-up gathered data on late neonatal mortality (death occurring between day 7 and day 30). This study included all BPL or ST women who had a livebirth and who were contacted at the three data collection points (n = 2919), which correspond to 89.1% of the original sample (n = 3273) (Fig. 1).

### Data Collection

Data collection was conducted between July 2013 and November 2014 by trained field workers. The main outcome NMR was defined as “the number of neonatal deaths per 1000 live births,” and a neonatal death was defined as any death within 28 days of life (Evaluation 2015). Maternal characteristics included: age, years of education, religion (Hindu, Muslim, and Christian), caste (Scheduled Tribe, Scheduled Caste, and Other), district of residency (Dahod, Sabarkantha, and Surendranagar), parity, number of antenatal care visits (ANC), and BPL status identified by a state issued BPL card.

Neonatal variables included age and gestational age. Expected delivery date was ascertained from the mother, and then gestational age at birth was computed by subtracting the expected delivery date from the actual delivery date. Gestational age was later coded into four categories: extremely/very premature (<28 to <32 weeks), moderately premature (32 to <37 weeks), term (37 to <42 weeks) and post-term neonates (42 or more weeks) (WHO 2014). Extremely and very premature categories were grouped together since there were few neonatal deaths in each category.

Data on childbirth characteristics collected from the mother included the following: place of delivery (home/on-the-way, public or private facilities), type of delivery (vaginal or C-sections), type of birth attendant (nurse, untrained personnel, doctor, gynecologist, and home/traditional birth attendant/self), and self-reported maternal complications during childbirth (excessive bleeding, high blood pressure, >12 h labor, mal-presentation, anemia, or convulsions).

### Analysis

Data was stored on an Electronic Data Capture (REDCap) database (Harris et al. 2009). Stata v12 (StataCorp, College Station, Texas) was used for analysis. Frequencies and percentages were used to describe the data and Pearson Chi square was used to assess bivariate differences between groups.

Multivariable logistic regression was used to obtain adjusted odds ratios (AOR) and 95% confidence intervals (CIs), and it was deemed to be appropriate since the incidence of the outcome was less than 10% (Katz 2011). Variables found to be significant at p < 0.20 in bivariate analyses were included in the multivariable models (Katz 2011). The variable birth attendants was considered an intermediate factor between place of delivery and neonatal mortality and was thus excluded from the multivariable models.

Multiplicative interaction was assessed between place of delivery and the other variables in the model, and significant interaction was found only with the variable for complications during childbirth (p < 0.05). The multivariable analyses are presented in two models: model 1 describes the adjusted association between neonatal deaths, maternal, child, and childbirth characteristics and model 2 contains an interaction term for place of delivery and maternal childbirth complications. Given the significant multiplicative interaction found in model 2, the association between neonatal mortality and place of delivery was further stratified by maternal complications during childbirth. We calculated the preventable fraction (1 minus the odds ratio) to determine the proportion of neonatal deaths that can be prevented if all mothers had an institutional rather than home delivery.

### Ethical Considerations

Ethical approval was granted by the Indian Institute of Public Health, Gandhinagar, Gujarat, India with the ethical approval number TRC-IEC No:23/2012 & KI: 2010/1671–31/5. I. All study participants completed written informed consent.

## Results

### Characteristics of the Study Participants

The mean maternal age was 24 years ± 3.41 (SD) with no significant differences between those reporting neonatal deaths and those that did not (data not shown). Thirty-eight percent lived in Sabarkantha, 33.6% in Dahod and the rest in Sabarkantha (Table 2). Forty eight percent of mothers had between two and three pregnancies. Ninety-six percent had a BPL card. Half of the neonates were male and 57.2% were born at term (Table 2).

**Table 2:** Neonatal mortality ratio by maternal and child characteristics, (n = 2919)

Maternal variables	Live births n = 2919	Live births (%)	Deaths n = 72	NMR	95% CI	p value^a^
Caste						
ST	996	34.1	23	23	15–34	0.78
SC	224	7.7	7	31	13–63	
Other	1699	58.2	42	25	18–33	
BPL card						
No	110	3.8	2	18	2–64	0.65
Yes	2809	96.2	70	25	3–31	
Parity						
Primipara	1068	36.6	25	23	15–34	0.65
2–3 pregnancies	1398	47.9	33	24	16–33	
4+ pregnancies	453	15.5	14	31	5–16	
District						
Dahod	980	33.6	38	39	28–53	<0.001
Sabarkantha	1134	38.8	19	17	10–26	
Surendranagar	805	27.6	15	19	10–30	
Sex of the baby						
Female	1369	46.9	34	25	17–35	0.96
Male	1550	53.1	38	25	17–33	
Gestational age						
Extremely/very preterm	69	2.4	8	116	51–216	<0.001
Moderate to late preterm	469	16.1	11	23	12–42	
Term	1671	57.2	41	25	18–33	
Post-term	710	24.3	12	17	9–29	

^a^Pearson Chi square test for significance between distributions among variables of interest

Eight in ten mothers delivered in an institution, and 93% had vaginal births. Nurses were the most common birth attendant (34.8%) (Table 3). Half reported having four or more antenatal care visits, and 11.7% reported an obstetric complication during delivery (Table 3).

**Table 3:** Neonatal mortality rate (NMR) by ANC and delivery characteristics (n = 2919)

Delivery variables	Live births	Live births (%)	Deaths	NMR	95% CI	p value^a^
Place of delivery						
Private	1787	61.2	42	24	17–32	0.73
Home/on way	489	16.8	15	31	17–50	
Public	643	22.0	15	23	13–38	
Type of delivery						
Vaginal	2724	93.3	65	24	18–30	0.29
C-section	195	6.7	7	36	15–73	
Birth attendant						
Nurse	1016	34.8	17	17	10–27	0.09
Untrained personnel	257	8.8	7	27	11–55	
General doctor	205	7.0	10	49	24–88	
Gynecologist	981	33.6	25	25	17–37	
Home/TBA/Self	460	15.8	13	28	15–48	
ANC visits						
Less than 3	1302	44.6	38	29	21–40	0.15
4 or more	1617	55.4	34	21	15–30	
Obstetric complication during delivery						
Yes	341	11.7	15	44	25–72	0.01
No	2578	88.3	57	22	17–28	

^a^Pearson Chi square test for significance between distributions among variables of interest

### Incidence of Neonatal Mortality in the Study Population

The overall NMR was 25 per 1000 live births (95% CI, 19–31) with the majority of deaths occurring during the first week of life, resulting in an early NMR of 19 per 1000 live births (95% CI, 14–24).

### Neonatal Mortality by Maternal, Child, and Delivery Characteristics

The bivariate analysis showed that NMR was significantly higher among neonates born to women living in the Dahod district, and among extremely/very preterm neonates (Table 2, p < 0.05).

In addition, neonates born to mothers with reported obstetric complications during delivery had a NMR twice higher than those born to mothers without childbirth complications (Table 3, p < 0.05). Of those women who reported a complication during delivery (n = 341), 79% delivered in a private obstetric care facility, 18.7% in a public facility and 2.3% at home/on the way (data not shown).

Place of delivery, caste/tribe, primiparity, sex of the baby, type of delivery, type of birth attendant, and number of ANC visits were not associated with higher NMRs (p > 0.05).

The three main causes of neonatal mortality were breathing complications or infection (48%), preterm/low birth weight complications (25%), and unclassifiable cases (20%). No associations were found between place of delivery and cause of neonatal death (p = 0.86, data not shown).

After adjustment by maternal self-reported obstetric complications during delivery, gestational age, ANC, and district, place of delivery was not associated with neonatal mortality (p > 0.05, model 1, Table 4). However, model 2 showed a significant multiplicative interaction between maternal self-reported complications during delivery and place of delivery (p < 0.05, Table 4). Institutional delivery was protective against neonatal mortality only among women with self-reported complications during delivery.

**Table 4:** Multivariable models without (model 1) and with (model 2) the interaction term for maternal complications during childbirth (n = 2919)

Variables	Model 1^a^	Model 2^a^	Stratification by maternal childbirth complications^a^	
	AOR (95% CI)	AOR (95% CI)	Yes (n = 341)	No (n = 2578)
Place of delivery				
Home	1.00	1.00	1.00	1.00
Public	0.74 (0.35–1.57)	0.69 (0.32–1.48)	0.03 (0.00–0.33)	1.00 (0.44–2.25)
Private	0.75 (0.40–1.40)	0.87 (0.46–1.66)	0.07 (0.01–0.45)	0.93 (0.47–1.85)
Maternal complications				
Yes	1.00	1.00		
No	0.41 (0.22–0.75)	0.04 (0.00–0.35)		
Interaction term^b^	–	0.46 (0.22–0.95)		

^a^Adjusted by gestational age, ANC, and district

^b^Interaction term equal to the product of place of delivery and maternal complications during childbirth

Among this group (n = 341), those who gave birth in a private or public facility had significantly lower odds of having a neonatal death than women delivering at home (AOR 0.07 95% CI 0.01–0.45 and AOR 0.03, 95% CI 0.00–0.33 respectively) (Table 4). We calculated the preventable fraction of neonatal deaths if all women reporting a complication (n = 341) delivered in a health institution. It showed that 97% of the neonatal deaths in this group could be prevented if all women gave birth in a public facility. The preventable fraction was similar if all mothers delivered at a private facility (93%).

## Discussion

Our community-based cohort study in a vulnerable population is the first study from India to report that institutional delivery was protective against neonatal mortality among neonates born to disadvantaged mothers with obstetric complications during delivery.

Neonatal mortality is a multi-causal phenomenon that is strongly linked to maternal health and skilled delivery (Lawn et al. 2005, 2010; Lee et al. 2011). Specifically, maternal complications during childbirth (preeclampsia, eclampsia, obstructed labor, and hemorrhage) have been highlighted as a key determinant of NMR around the world (Lawn et al. 2005, 2010; Zupan 2005).

Institutional delivery might decrease NMR through several pathways. Delivering in a facility can increase the odds of detecting maternal complications, which enables adequate and opportune treatment of mothers and thus protection of neonates. Maternal complications during delivery have been reported as a key factor in neonatal mortality in India (Kumar et al. 2014). Thus, our finding that there is a protective effect of institutional delivery on NMRs among disadvantaged mothers with complications during delivery highlights the relevance of improvements to emergency obstetric care quality and accessibility in order to further decrease NMR. These key findings are also in accordance with an Indonesian study showing similar results, albeit among urban populations (Titaley et al. 2012).

Our results stress that Gujarat government´s emphasis on promoting institutional delivery among BPL or ST mothers (De Costa et al. 2014) is a step in the right direction, not just for maternal death reduction but also for NMR reduction. Given the concentration of both these outcomes in poor women, focused emphasis on getting them into health facilities for delivery is a worthwhile one. Maternal complications during childbirth are often unpredictable (Ronsmans and Graham 2006) thus efforts to improve women’s access to skilled delivery must continue in this setting. This is particularly relevant given that intra-partum risk factors have been acknowledged as more important for neonatal mortality than those identified during pregnancy (Lawn et al. 2005).

In our study, institutional deliveries at both public and private facilities were protective against neonatal mortality. Our data indicating that delivering in a private facility was protective against neonatal mortality contrast with a study by Kumar et al. conducted in the Bihar state India, which found the opposite (Kumar et al. 2014). This disparity might reflect differences in the quality of the private obstetric care available between states. It is possible that in a richer state such as Gujarat (Ram et al. 2013), mothers can afford to use higher quality private providers than in Bihar, which is a relatively poor state (Ram et al. 2013). Nevertheless, our findings showing the protective effect of private institutional delivery are in line with those reported by a multi-country study conducted in 67 low- and middle-income countries (Fink et al. 2015).

Previous studies have reported that delivering a public facility was not a protective factor against early neonatal mortality (Fink et al. 2015; Titaley et al. 2012), and one found that delivering in a public hospital increased the odds of having a neonatal death (Titaley et al. 2012). However, our findings suggest that delivering in public facility was protective against neonatal mortality even though it has been argued that limited access to emergency obstetric/neonatal care could be reasons behind the lack of association between public facility delivery and higher neonatal survival in other settings (Titaley et al. 2012). One possible explanation is that in our study, most complicated deliveries in the public sector were treated at community health centers or hospitals that are expected to have enough resources to curtail NMR. However, more studies are needed to assess whether this is true or not.

A final possible pathway by which institutional delivery can decrease neonatal mortality is through increasing timely access to specialized neonatal care. As our study did not focus on the role that specialized care had on neonatal survival, further qualitative and quantitative studies are necessary to clarify this relationship.

### Limitations

A limitation of our study is that our variable on delivery complications was self-reported which can lead to underestimation of the incidence of obstetric complications during delivery in this setting. In addition, there is a risk of misclassification of early neonatal deaths as stillbirths, which could have resulted in underestimation of the NMRs reported in this study. Geographic generalizability to the state and the country is limited because of heterogeneity in the population subgroups and health care provision levels.

## Conclusions

Our findings highlight the vital role of institutional delivery in preventing neonatal mortality among newborns of vulnerable (BPL or tribal) women who experienced complications during delivery in this setting. Efforts to improve vulnerable women’s access to obstetric care are critical in this setting if NMRs are to be further decreased.

## References

[CR1] Bharadwaj, M. (2011). Provisional population totals: Rural–urban distribution Gujarat 2011. Retrieved September 21, 2015 from http://censusgujarat.gov.in/Downloads/PPT/Paper-2/Paper-2 Volume-1 of 2011 Gujarat.pdf.

[CR2] Darmstadt GL, Bhutta ZA, Cousens S, Adam T, Walker N, de Bernis L (2005). Evidence-based, cost-effective interventions: how many newborn babies can we save?. Lancet.

[CR3] De Costa A, Vora KS, Ryan K, Sankara Raman P, Santacatterina M, Mavalankar D (2014). The state-led large scale public private partnership ‘Chiranjeevi Program’ to increase access to institutional delivery among poor women in Gujarat, India: How has it done? What can we learn?. PLoS ONE.

[CR4] Dogra V, Khanna R, Jain A, Kumar AMV, Shewade HD, Majumdar SS (2015). Neonatal mortality in India’s rural poor: Findings of a household survey and verbal autopsy study in Rajasthan, Bihar and Odisha. Journal of Tropical Pediatrics.

[CR5] Feng XL, Guo S, Hipgrave D, Zhu J, Zhang L, Song L, Ronsmans C (2011). China’s facility-based birth strategy and neonatal mortality: A population-based epidemiological study. Lancet.

[CR6] Fink G, Ross R, Hill K (2015). Institutional deliveries weakly associated with improved neonatal survival in developing countries: Evidence from 192 demographic and health surveys. International Journal of Epidemiology.

[CR7] Government of Gujarat (2009). Annual Administrative Report, 2007–2008. Retrieved September 22, 2015 from http://www.gujhealth.gov.in/images/pdf/aar07-08.pdf.

[CR8] Harris PA, Taylor R, Thielke R, Payne J, Gonzalez N, Conde JG (2009). Research electronic data capture (REDCap): A metadata-driven methodology and workflow process for providing translational research informatics support. Journal of Biomedical Informatics.

[CR9] Katz M (2011). Multivariable analysis: A practical guide for clinicians and public health researchers.

[CR10] Kumar G, Dandona R, Chaman P, Singh P, Dandona L (2014). A population-based study of neonatal mortality and maternal care utilization in the Indian state of Bihar. BMC Pregnancy and Childbirth.

[CR11] Lawn JE, Cousens S, Zupan J (2005). 4million neonatal deaths: When? Where? Why?. Lancet.

[CR12] Lawn JE, Kerber K, Enweronu-Laryea C, Cousens S (2010). 3.6million neonatal deaths: What is progressing and what is not?. Seminars in Perinatology.

[CR13] Lee A, Cousens S, Darmstadt G, Blencowe H, Pattinson R, Moran N, Lawn J (2011). Care during labor and birth for the prevention of intrapartum-related neonatal deaths: A systematic review and Delphi estimation of mortality effect. BMC Public Health.

[CR14] Limwattananon S, Tangcharoensathien V, Sirilak S (2011). Trends and inequities in where women delivered their babies in 25 low-income countries: Evidence from Demographic and Health Surveys. Reproductive Health Matters.

[CR15] Measure Evaluation. (2015). Family planning and reproductive health indicators database-neonatal mortality rate (NMR). Retrieved September 23, 2015 from http://www.cpc.unc.edu/measure/prh/rh_indicators/specific/nb/neonatal-mortality-rate-nmr.

[CR16] Ministry of Social Justice and Empowerment. State-wise percentage of population below poverty line by social groups, 2004–2005. Retrieved September 22, 2015 from http://socialjustice.nic.in/socialg0405.php.

[CR17] Paul VK, Sachdev HS, Mavalankar D, Ramachandran P, Sankar MJ, Bhandari N, Kirkwood B (2011). Reproductive health, and child health and nutrition in India: Meeting the challenge. Lancet.

[CR18] Ram U, Jha P, Ram F, Kumar K, Awasthi S, Shet A, Kumar R (2013). Neonatal, 1–59month, and under-5 mortality in 597 Indian districts, 2001 to 2012: Estimates from national demographic and mortality surveys. The Lancet Global Health.

[CR19] Rammohan A, Iqbal K, Awofeso N (2013). Reducing neonatal mortality in India: Critical role of access to emergency obstetric care. PLoS ONE.

[CR20] Ronsmans C, Graham WJ (2006). Maternal mortality: Who, when, where, and why. Lancet.

[CR21] Sidney K, de Costa A, Diwan V, Mavalankar DV, Smith H (2012). An evaluation of two large scale demand side financing programs for maternal health in India: The MATIND study protocol. BMC Public Health.

[CR22] The Million Death Study Collaborators (2006). Causes of neonatal and child mortality in India: A nationally representative mortality survey. Lancet.

[CR23] Titaley CR, Dibley MJ, Roberts CL (2012). Type of delivery attendant, place of delivery and risk of early neonatal mortality: Analyses of the 1994–2007 Indonesia demographic and health surveys. Health Policy and Planning.

[CR24] Tura G, Fantahun M, Worku A (2013). The effect of health facility delivery on neonatal mortality: Systematic review and meta-analysis. BMC Pregnancy and Childbirth.

[CR25] Wardlaw, T., Amouzou, A., Velez, L., Dwivedi, A., & Hug, L. (2014). Committing to child survival: A promise renewed. Progress Report 2014. Retrieved September 21, 2015 from http://www.apromiserenewed.org/APR_2014_web_15Sept14.pdf.

[CR26] WHO (2014). Preterm birth. Fact sheet No. 363: Retrieved September 22, 2015 from http://www.who.int/mediacentre/factsheets/fs363/en/.

[CR27] World Health Organization (2003). Integrated Managemnet of Neonatal and childhood illnesses.

[CR28] You, D., Hug, L., Chen, Y., Newby, H., & Wardlaw, T. Levels and Trends in Child Mortality (2014). Retrieved September 22, 2015 from http://www.unicef.org/media/files/Levels_and_Trends_in_Child_Mortality_2014.pdf.

[CR29] Zodpey, S., & Paul, V. (2014). State of India’s newborns (SOIN) 2014-a report. Retrieved September 22, 2015 from http://www.newbornwhocc.org/SOIN_PRINTED 14-9-2014.pdf.

[CR30] Zupan J (2005). Perinatal mortality in developing Countries. New England Journal of Medicine.

